# Immune cells and the trajectories of depression, anxiety, and cognitive function among people with amyotrophic lateral sclerosis

**DOI:** 10.1016/j.bbih.2024.100907

**Published:** 2024-11-22

**Authors:** Yihan Hu, Elie Deeba, Ulf Kläppe, Linn Öijerstedt, John Andersson, Nicolas Ruffin, Fredrik Piehl, Caroline Ingre, Fang Fang, Christina Seitz

**Affiliations:** a Institute of Environmental Medicine, Karolinska Institutet, Stockholm, Sweden; b Department of Neurology, Karolinska University Hospital, Stockholm, Sweden; c Department of Clinical Neuroscience, Karolinska Institutet, Stockholm, Sweden

**Keywords:** Amyotrophic lateral sclerosis, Leukocytes, T cells, Anxiety, Depression, Cognitive function

## Abstract

**Background:**

Amyotrophic lateral sclerosis (ALS) represents a complex syndrome characterized by motor, psychiatric, and cognitive symptoms, where associations between cellular immune features and non-motor manifestations remain unknown.

**Methods:**

In this cohort study, we enrolled 250 incident people with ALS (pwALS) assessed with the Hospital Anxiety and Depression Scale, and 226 pwALS with the Montreal Cognitive Assessment, including 218 overlapping pwALS. All individuals were diagnosed between January 2015 and January 2023 in Stockholm, Sweden. We applied joint latent class models to delineate distinct trajectories of anxiety, depression, and cognition, incorporating survival outcomes. A majority of the pwALS had data on leukocyte counts and flow cytometric analyses using a comprehensive T cell panel. We then used immune cell subtypes measured at diagnosis to predict trajectories of these outcomes following ALS diagnosis.

**Results:**

We identified two distinct trajectories for anxiety, depression, and cognitive function following ALS diagnosis. PwALS with longer survival displayed more stable trajectories, while those with shorter survival showed decreasing anxiety symptom, increasing depressive symptom, and declining cognitive function. Higher count of leukocytes at the time of ALS diagnosis tended to associate with anxiety and depression trajectories related to shorter survival. Among T cell subpopulations, several CD8^+^ T cell subsets were associated with a stable trajectory of depressive symptom, and, in turn, better survival.

**Conclusion:**

ALS-associated psychiatric and cognitive trajectories vary significantly between pwALS with different prognosis. Certain T cell subsets measured at diagnosis might be indicative of depression trajectories post-diagnosis.

## Highlights

• People with ALS who survive longer tend to have stable mental states.
• Higher leukocyte counts are correlated with unstable psychiatric states.
• Some CD8^+^ T cell subsets are correlated with better depressive outcomes.

## 1 Background

Amyotrophic lateral sclerosis (ALS) is a devastating neurodegenerative disease that primarily affects upper and lower motor neurons, leading to progressive muscle weakness (Feldman et al., 2022). The two most common presentations of ALS are bulbar and spinal onset. People with ALS (pwALS) with bulbar onset typically experience initial symptoms in the form of impaired tongue movement, slurry speech, and problems with swallowing, while spinal-onset ALS presents with asymmetrical limb weakness (Hardiman et al., 2017). Notably, bulbar-onset ALS tends to progress more rapidly with a lower survival rate compared to spinal-onset ALS (Feldman et al., 2022). While age, sex, and certain genetic mutations have been linked to these variations, the full spectrum of factors influencing these differences remains largely unknown (Chiò et al., 2020). ALS is increasingly acknowledged as a syndrome with additional non-motor manifestations, including psychiatric and cognitive symptoms (Goutman et al., 2022). Approximately 35%–45% of pwALS display mild to moderate cognitive impairment, while about 14% fulfill criteria for dementia, predominantly frontotemporal dementia (FTD)(Pender et al., 2020). Psychiatric symptoms are also prevalent among pwALS, with about a third developing depression, although prevalence of anxiety is less clear (Heidari et al., 2021; Kurt et al., 2007).

Emerging evidence underscores a complex interaction between the brain's resident immune system and peripheral immune cells, potentially contributing to the pathophysiology of psychiatric disorders and cognitive impairment (Bennett and Molofsky, 2019; Zhao et al., 2022). This interaction involves both the innate and adaptive immune systems, as has been shown in depression, schizophrenia, bipolar disorder, and cognitive impairment (Branchi et al., 2021; Khandaker and Dantzer, 2016; Benedetti et al., 2020; Farina et al., 2022). The CNS is traditionally considered immune-privileged; however, it is now understood that peripheral immune cells are actively involved in processes like neuroprotection and synaptic plasticity (Zhao et al., 2022; Toben and Baune, 2015). Lymphocytes, known for their functional diversity, are critical in determining the specificity of immune responses. Research conducted in experimental mouse models suggests that absence of mature T cells is associated with impairment of cognitive functions, which can be reversed by passive T-cell transfer (Kipnis et al., 2004). Additionally, stress-induced neuroinflammation can lead to maladaptive T-cell responses, potentially aggravating neurodegeneration (Toben and Baune, 2015). Furthermore, alterations in Th1-like cell-mediated and Th2-like antibody-associated immune responses have been observed in psychiatric disorders and Alzheimer's disease (Schwarz et al., 2001). The involvement of immune responses in neurodegenerative diseases, psychiatric disorders, and cognitive dysfunction might therefore contribute to the higher-than-expected concurrence of these conditions (Shaw et al., 2023).

The objective of this study was to explore associations between immune parameters and psychiatric and cognitive outcomes among newly diagnosed pwALS, focusing on: 1) Assessing the temporal trajectories for anxiety, depression, and cognitive function; 2) Determining correlations between cellular immune markers and measures of anxiety, depression, and cognitive function; and 3) Exploring the predictive role of these immune markers for trajectories of psychiatric and cognitive measures.

## 2 Methods

### 2.1 Data sources

**Fig. 1:**
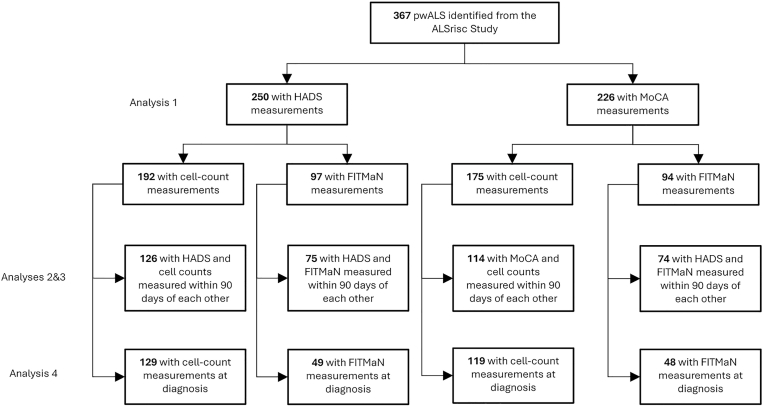
Flowchart of the study design.

In this study, incident cases of pwALS were recruited from the ALSrisc Study (Chourpiliadis et al., 2024), a case-control study in Stockholm, Sweden. All the pwALS received a diagnosis of possible, probable, or definite ALS, according to the revised El Escorial criteria. A modified version of the Hospital Anxiety and Depression Scale (HADS) tailored for ALS (i.e., after removing the original item eight and item eleven)(Gibbons et al., 2011) and the Montreal Cognitive Assessment (MoCA) were used in the ALSrisc Study to screen for symptoms of anxiety and depression and cognitive function, respectively, at the time of diagnosis and approximately every six months thereafter (Chourpiliadis et al., 2024). Additional data was collected through the Swedish Motor Neuron Disease (MND) Quality Registry, which was established in 2015 and covers approximately 85% of all MND patients in Sweden, although the coverage in the Region of Stockholm approaches 99% (Longinetti et al., 2018). The registry gathers a wide array of data, encompassing clinical scales, patient-reported outcomes, and selected biomarkers. A total of 367 pwALS included in the ALSrisc Study were diagnosed between January 2015 and January 2023. Due to data availability, 250 pwALS were included in the cohort analyzing HADS (i.e., HADS cohort) and 226 in the cohort analyzing MoCA (i.e., MoCA cohort), with 218 pwALS who were included in both (Fig. 1). We followed all patients from date of diagnosis until death or end of study (January 6, 2023), whichever came first.

### 2.2 Immune measurements

The procedure for blood sample collection and analysis has been described previously (Cui et al., 2022). In brief, freshly collected blood samples were processed within 24 h to measure leukocytes and T cells, following validated protocols at the Departments of Clinical Chemistry and Clinical Immunology and Transfusion Medicine, Karolinska University Hospital, respectively. The analysis of leukocyte counts included neutrophils, lymphocytes, monocytes, eosinophils, and basophils. The latter two were excluded from the analysis of the present study as their numbers were often below the lower limit of detection. We then conducted flow cytometric analyses using the comprehensive T lymphocyte panel FITMaN (Flow Immunophenotyping Technical Meeting at NIH) (Maecker et al., 2012) to evaluate frequencies of the following T cell subsets: CD3^+^ T cells, CD4^+^ T cells, CD8^+^ T cells, double positive (DP) CD4^+^CD8^+^ T cells, and double negative (DN) CD4^−^CD8^−^ T cells. Furthermore, we assessed memory phenotypes of CD4^+^ and CD8^+^ T cells based on CCR7 and CD45RA expression, including CCR7^+^CD45RA^+^ naïve T cells, CCR7^+^CD45RA^−^ central memory T cells (T_CM_), CCR7^-^ CD45RA^−^ effector memory T cells (T_EM_), and CCR7^−^CD45RA^+^ effector memory cells re-expressing CD45RA T cells (T_EMRA_). Finally, we measured the frequencies of CXCR3^+^CCR6^-^ Th1 cells, CXCR3^+^CCR6^+^ Th1/17 T cells, CXCR3^−^CCR6^-^ Th2 cells, and CXCR3^−^CCR6^+^ Th17 cells within the CD4^+^ T_CM_ and CD4^+^ T_EM_ cells.

C-reactive protein (CRP) was also measured at the Department of Clinical Chemistry, Karolinska University Hospital, according to established protocols.

### 2.3 Statistical analysis

The present study consists of four analyses. In all analyses, the measures of anxiety and depressive symptoms as well as cognitive function were analyzed as continuous variables, whereas the leukocyte counts and frequencies of T cell subsets underwent normalization through the Adaptive Cox-Box (ABC) transformation, followed by standardization (Yu et al., 2022). The ABC transformation accommodates a wide range of distribution types.

In Analysis 1, we used joint latent class model to investigate the longitudinal changes of psychiatric symptoms (anxiety and depression) and cognitive function in the HADS cohort and MoCA cohort, respectively (Kyheng et al., 2021). This model is composed of three sub-models: A) a multinomial logistic regression to stratify pwALS into homogenous groups based on the trajectories of psychiatric/cognitive measures and survival outcome; B) a linear mixed model to assess longitudinal dynamics of psychiatric/cognitive measures; and C) a survival model (class specific Weibull hazard) that focuses on time-to-event, i.e., survival. This comprehensive approach facilitates the identification of pwALS with different trajectories of psychiatric/cognitive measures and addresses the issue of informative censoring, i.e., those with faster disease progression are followed for a shorter time. The logistic regression sub-model (A), determining class membership probability, was formulated without incorporating covariates. In contrast, adjustment for age at diagnosis, sex, site of onset (bulbar vs non-bulbar), and time since diagnosis was used in the mixed model (B), and age at diagnosis, sex, and site of onset were adjusted for in the survival model (C). The determination of the optimal number of latent classes was guided by the Bayesian Information Criterion and clinical interpretability.

In Analysis 2, we focused on the pwALS who had at least one measurement of immune cells and HADS/MoCA conducted within ±90 days of each other. We used the first measurement of immune cells and HADS/MoCA for each patient to evaluate the correlation between different immune cells (i.e., leukocytes cell counts and T cell subsets frequencies) and anxiety, depression, and cognitive function. This analysis was conducted using a linear regression model, adjusted for age at diagnosis, sex, and site of onset.

In Analysis 3, we expanded Analysis 2, by encompassing all measurements of immune cells and HADS/MoCA to understand if the correlations noted with the first measurements also persisted during follow-up. This analysis was conducted using linear mixed models with both random slope and random intercept. We similarly evaluated the correlations between immune cells and anxiety, depression, and cognitive function, adjusting for the same covariates as in Analysis 2.

In Analysis 4, we utilized immune cell measurements within ±90 days of diagnosis to model their predictive value for the psychiatric and cognitive trajectories identified in Analysis 1. PwALS were classified as fast or slow progressors based on the latent class label of Analysis 1, considering the trajectories of anxiety, depression, and cognitive function separately. We then applied logistic regression, adjusting for the same covariates as in Analysis 2, to assess the odds ratio (OR) of belonging to the fast progressor group in relation to the immune cells at the time of diagnosis. Considering the potential fluctuations in immune cell measurements due to inflammatory conditions, such as infections and autoimmune diseases, we performed a sensitivity analysis after excluding pwALS who had a level of CRP above 10 mg/L, which indicates clinically evident inflammation, within ±30 days of their immune cell measurement.

## 3 Results

Among the 250 and 226 pwALS included in the HADS and MoCA cohorts, respectively, 192 (76.8%) and 175 (77.4%) had measurements on immune cells (Table 1). The mean age at diagnosis in both cohorts was approximately 65 years, with a slightly higher proportion of males compared to females. As expected, given the substantial overlap between the two cohorts, there was no significant difference in age at diagnosis, sex, site of onset, scores on the Amyotrophic Lateral Sclerosis Functional Rating Scale - Revised (ALSFRS-R), or disease progression rate. No great difference was noted between the sub-cohorts with immune cell measurements and their main cohorts, either.

**Table 1:** Characteristics of pwALS included in the analysis.

Characteristics	Total (n = 367)	HADS (n = 250)	HADS & cell count (n = 192)	MoCA (n = 226)	MoCA & cell count (n = 175)
Age at diagnosis, mean (SD)	66.3 (11.0)	64.9 (10.9)	64.8 (10.6)	64.8 (10.9)	64.9 (10.6)
Sex, n. (%)					
Female	172 (46.9)	119 (47.6)	89 (46.4)	104 (46.0)	79 (45.1)
Male	195 (53.1)	131 (52.4)	103 (53.6)	122 (54.0)	96 (54.9)
Site of onset, n. (%)					
Bulbar	134 (36.5)	84 (33.6)	68 (35.4)	78 (34.5)	61 (34.9)
Non-bulbar	233 (63.5)	166 (66.4)	124 (64.6)	148 (65.5)	114 (65.1)
ALSFRS-R at diagnosis, mean (SD)	37.5 (7.4)	38.4 (6.6)	38.3 (6.7)	39.4 (5.5)	39.3 (5.5)
Progression rate, mean (SD)	0.9 (0.8)	0.8 (0.7)	0.8 (0.7)	0.7 (0.7)	0.7 (0.7)
Progression rate, n. (%)					
<0.5	84 (22.9)	78 (31.2)	76 (39.6)	75 (33.2)	74 (42.3)
≥0.5 and < 1.1	77 (21.0)	59 (23.6)	58 (30.2)	56 (24.8)	55 (31.4)
≥1.1	57 (15.5)	41 (16.4)	41 (21.4)	31 (13.7)	31 (17.7)
Unknown	149 (40.6)	72 (28.8)	17 (8.9)	64 (28.3)	15 (8.6)

HADS: Hospital Anxiety and Depression Scale; MoCA: Montreal Cognitive Assessment; ALSFRS-R: Amyotrophic Lateral Sclerosis Functional Rating Scale-revised; SD: standard deviation.

**Fig. 2:**
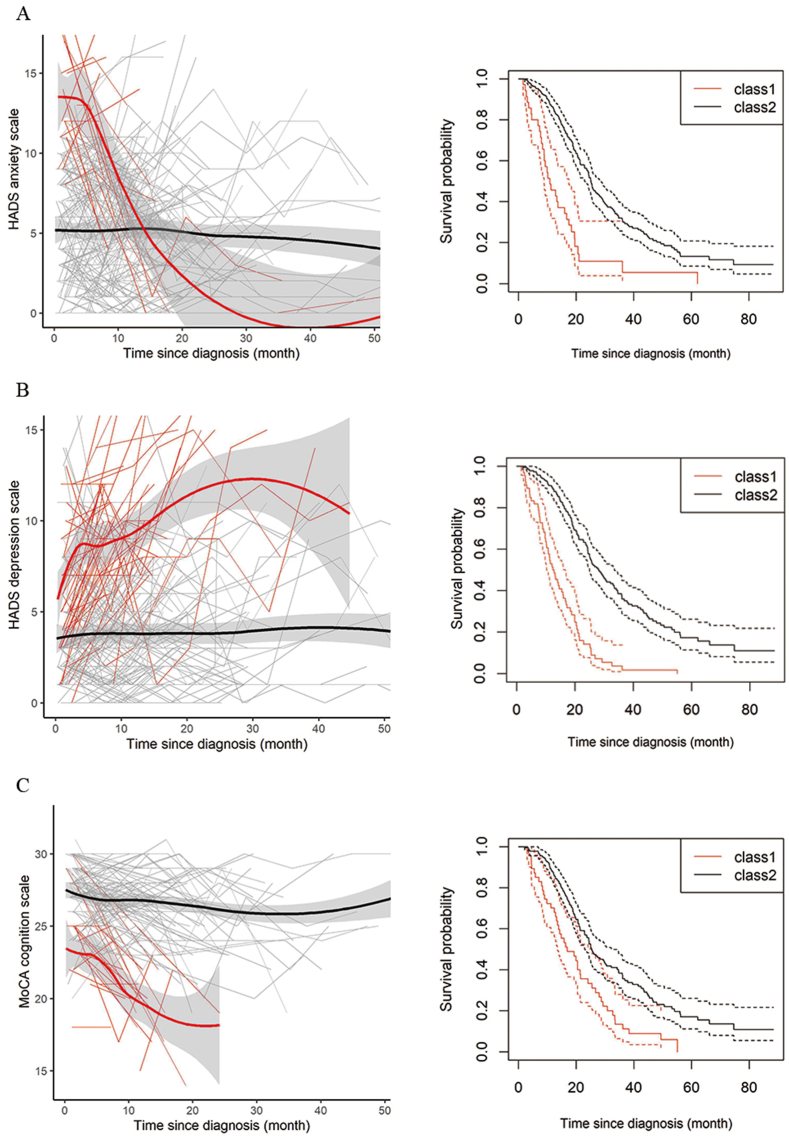
Latent groups by temporal trajectories of anxiety (A), depression (B), and cognition (C) as well as the respective survival probability of each group.

The first analysis identified two latent groups with differential trajectories of anxiety, depression, and cognition, respectively (Fig. 2). In terms of anxiety, the majority of pwALS (n = 215; 86.0%) displayed a stable trajectory, with a monthly rate of change of 0.01 (95% CI, −0.01 to 0.02), whereas a minority (n = 35; 14.0%) had declining scores at a monthly rate of −0.24 (95% CI, −0.32 to −0.17) (Fig. 2A). The stable group had an anxiety score that was 6.2 points lower at diagnosis compared to the declining group (*p* for difference <0.001). A similar pattern, but in the opposite direction, was evident for depression; a stable group (n = 184; 73.6%, monthly rate of change 0.02 [95%CI, 0.00 to 0.03]) and a group with increasing scores (n = 66; 26.4%, monthly rate of change 0.30 [95%CI, 0.20 to 0.41]) (Fig. 2B). At baseline, the depression score was 2.4 points lower in the stable group compared to the increasing group (*p* for difference = 0.004). For cognition, a majority (n = 179; 79.2%) had a stable trajectory (monthly rate of change −0.05 [95%CI, −0.07 to −0.03]) while a minority (n = 47; 20.8%) displayed a declining function (monthly rate of change −0.38 [95%CI, −0.48 to −0.28]) (Fig. 2C). The baseline cognitive function score was 2.3 points higher in the stable group compared to the declining group (*p* for difference <0.001). The stable and deviating trajectories were notably also associated with survival probability, as pwALS with longer survival were more likely to be in the stable groups (*p* for difference <0.001 in all analyses).

In the second analysis, we found no clear evidence of correlation between leukocyte counts and anxiety, depression, or cognitive function measured within ±90 days of each other, focusing on the first measurements (Table 2). However, among T cell subsets, a negative correlation was suggested between Th2_CM_ cell frequency and anxiety (*p* = 0.05), whereas a positive correlation was noted between CD4^+^ T_CM_ cell frequency and depression (*p* = 0.02) (Supplementary Table 1).

**Table 2:** Correlations between counts of leukocytes and mental health outcomes using data from first measurements^a^.

Cell type	Anxiety			Depression			Cognition		
	estimate	se	*p*	estimate	se	*p*	estimate	se	*p*
Leukocytes	−0.03	0.14	0.81	−0.16	0.12	0.20	0.12	0.12	0.31
Neutrophils	0.02	0.17	0.89	−0.14	0.14	0.34	0.11	0.13	0.43
Lymphocytes	−0.52	0.44	0.24	−0.51	0.37	0.17	0.39	0.52	0.45
Monocytes	0.91	1.86	0.63	0.14	1.55	0.93	1.62	1.54	0.29

se: standard error.

a Derived from linear regression, adjusted for age at diagnosis, sex, and site of onset.

The third analysis, incorporating all measurements of immune cells and mental health outcomes collected throughout follow-up did still not reveal evidence of a clear correlation (Table 3). However, a negative correlation was observed between Th2_CM_ cell frequency and anxiety (*p* = 0.02), along with a positive correlation between the lymphocyte marker CD45^+^ and depression (*p* = 0.04) (Supplementary Table 2).

**Table 3:** Correlations between counts of leukocytes and mental health outcomes using data from repeated measurements^a^.

Cell type	Anxiety			Depression			Cognition		
	estimate	se	*p*	estimate	se	*p*	estimate	se	*p*
Leukocytes	0.08	0.12	0.47	0.00	0.11	0.97	0.04	0.11	0.70
Neutrophils	0.15	0.13	0.23	0.05	0.12	0.67	−0.01	0.13	0.92
Lymphocytes	−0.50	0.43	0.24	−0.56	0.37	0.13	0.53	0.49	0.29
Monocytes	1.19	1.52	0.44	1.73	1.36	0.21	2.41	1.45	0.10

se: standard error.

a Derived from linear mixed model with random intercept and random slope, adjusted for age at diagnosis, sex, and site of onset.

In the final analysis, we found that a higher count of most leukocyte subtypes measured at diagnosis was associated with a greater risk of belonging to a trajectory for anxiety and depression, but not cognition, associated with poorer survival probability (Table 4). After excluding pwALS who had a CRP level above 10 mg/L around the time of immune cell measurement, we observed similar results, although statistically significant result was only noted for anxiety (Supplementary Table 3). Among distinct T cell subsets, higher frequencies of naïve CD8^+^ T cells, CD8^+^ T_CM_ cells, and CD8^+^ T_EM_ cells at time of diagnosis were associated with greater probability of belonging to the stable trajectory of depression, while higher frequencies of Th17_CM_ cells were associated with greater probability of belonging to the stable trajectory for cognitive function (Supplementary Table 4).

**Table 4:** Association between counts of leukocytes at the time of diagnosis and risk of demonstrating a negative trajectory of mental health outcomes during follow-up^a^.

Cell type	Odds Ratio (95% Confidence Interval)		
	Anxiety	Depression	Cognition
Leukocytes	1.91 (0.83–4.39)	1.16 (0.61–2.20)	0.95 (0.47–1.94)
Neutrophils	1.71 (0.72–4.07)	0.95 (0.50–1.80)	0.95 (0.48–1.91)
Lymphocytes	1.47 (0.72–3.01)	1.51 (0.88–2.61)	0.90 (0.51–1.62)
Monocytes	2.31 (0.82–6.45)	1.42 (0.68–2.99)	0.64 (0.29–1.42)

se: standard error.

a Derived from logistic regression, adjusted for age at diagnosis, sex, and site of onset.

## 4 Discussion

In this cohort study of incident cases of pwALS, we identified subgroups with stable or changing status of anxiety, depression, and cognitive function over time after diagnosis. Notably, individuals with a more stable psychiatric and cognitive profile demonstrated a better survival, whereas those with poorer survival displayed diminishing anxiety symptom, increasing depressive symptom, and declining cognitive function over time.

Levels of anxiety and depression were previously reported as stable in pwALS (Goldstein et al., 2006; Thakore and Pioro, 2016). However, this observation may be biased due to higher response rates of individuals with slower disease progression, since pwALS with unstable mental state or faster progression might contribute less observations. In contrast, our study revealed distinct trajectories for anxiety and depression among pwALS with shorter survival, compared to pwALS with longer survival. The opposite direction of change in anxiety and depression likely reflects the emergence of apathy among some of the pwALS (Kutlubaev et al., 2023).

Our findings on cognition are in line with previous research suggesting that pwALS with impaired cognitive function at diagnosis show a continued decline in cognition over time, whereas those with normal cognitive function at diagnosis tend to remain cognitively intact (Elamin et al., 2013; Consonni et al., 2021). However, this likely also depends on the instrument used to measure cognitive function. For example, using a modified version of the Edinburgh Cognitive and Behavioral ALS Screen (KS-ECAS), there was little difference in the trajectories between psALS with normal or impaired cognition at diagnosis (Öijerstedt et al., 2024).

When exploring the correlations between immune cells and psychiatric and cognitive outcomes measured at ALS diagnosis or during follow-up, no clear pattern was identified. However, higher counts of major leukocyte subsets at the time of diagnosis were observed to indicate a greater probability of belonging to the groups displaying deviating trajectories for anxiety and depression (i.e., groups with shorter survival). A possible explanation is that anxiety and depression can induce stress, which may lead to leukocyte elevation by enhancing the innate immune response through the mobilization of neutrophils and monocytes in the periphery (Ishikawa and Furuyashiki, 2022; Nishitani and Sakakibara, 2014). These cells can infiltrate the brain's perivascular space, release cytokines, and affect vascular endothelial functions, thereby further exacerbating the anxiety and depressive symptoms (Ishikawa and Furuyashiki, 2022). Several studies have shown an increase in pro-inflammatory cytokines, such as IL-6 and IL-10, among pwALS (Hu et al., 2017; Tortelli et al., 2020). These pro-inflammatory cytokines can also contribute to depression or anxiety by inducing oxidative and nitrosative stress, causing brain damage, and reducing serotonin availability (Catena-et al., 2011).

We observed that an increased frequency of some T cell subsets, e.g., naïve CD8^+^ T cells, measured at the time of ALS diagnosis had a negative association with the risk of demonstrating a trajectory of increasing depressive symptom and poorer survival. The increased frequency of naïve CD8^+^ T cells may suggest their presence in a quiescent state, indicative of a less inflammatory environment (Sprent and Surh, 2011). We also found that a higher frequency of Th17_CM_ cells at diagnosis was associated with greater likelihood of a stable trajectory of cognitive function. This finding is in contrast to previous studies showing that a higher level of peripheral Th17 cells is associated with lower cognitive function (Lu et al., 2022; Cipollini et al., 2019). The contrasting findings could be due to different reasons; however, we have to note that the previous studies focused on the Th17 cell population as a whole, whereas in the present study, we studied different Th17 subpopulations.

Strengths of our study include the inclusion of newly diagnosed pwALS and the possibility to leverage data through the Swedish MND Quality Registry. The integration of survival data to the longitudinal assessment of psychiatric symptoms and cognitive function over time mitigated the bias stemming from informative censoring, i.e., pwALS with a faster progression contributed less information than pwALS with a slower progression.

The study also has several limitations. First, there is some bias towards slower disease progression among the sub-cohorts with available data on immune cells, compared to their corresponding main cohorts, which may have impacted the results on correlations between immune cells and psychiatric and cognitive outcomes. Second, immune cells were only assessed in blood and may not reflect the immune responses of the central nervous system. In a previous study, we found relatively weak correlations between T cell subsets measured in blood and cerebrospinal fluid (Yazdani et al., 2022, 2024). Third, levels of immune cells fluctuate, e.g., due to infections, autoimmune diseases and allergies, or use of immune-modulating medications including corticosteroids. The similar results obtained after excluding pwALS with a CRP indicative of clinically evident inflammation helped to alleviate such concern to some extent, but not completely. Fourth, MoCA may not detect subtle cognitive changes whereas motor function impairment in pwALS could also affect the performance of tasks in MoCA. Further, given the high degree of correlations between different immune cell populations and the exploratory nature of this study, we did not use standard correction methods for multiple testing (e.g., false discovery rate). Regardless, findings of this study must be interpreted with caution and require validation from independent studies. Finally, given the Stockholm-based study sample, the results of this study may not be generalizable to the entire Sweden or to other countries.

In conclusion, we observed significantly varying psychiatric and cognitive trajectories among pwALS with different prognoses. PwALS with worse prognoses showed associations with higher numbers of leukocyte subsets and specific T cell subsets.

## CRediT authorship contribution statement

**Yihan Hu:** Writing – original draft, Methodology, Formal analysis, Conceptualization. **Elie Deeba:** Writing – review & editing, Writing – original draft. **Ulf Kläppe:** Writing – review & editing. **Linn Öijerstedt:** Writing – review & editing. **John Andersson:** Writing – review & editing. **Nicolas Ruffin:** Writing – review & editing. **Fredrik Piehl:** Writing – review & editing. **Caroline Ingre:** Writing – review & editing, Resources, Project administration. **Fang Fang:** Writing – review & editing, Resources, Project administration, Methodology, Conceptualization. **Christina Seitz:** Writing – review & editing, Writing – original draft, Supervision, Project administration, Methodology, Investigation, Formal analysis, Conceptualization.

## Ethics approval and consent to participate

The study was approved by the Swedish Ethical Review Authority (DNRs: 2014/1815-31/4, 2018-1065/31, and 2021-06397-02). The reporting of data complies with the STROBE guidelines for cohort study. Informed consent has been obtained from all participants of the study.

## Consent for publication

Not applicable.

## Role of the funder/sponsor

The funders had no role in the design and conduction of the study, writing of the manuscript, or decision to submit for publication.

## Funding

The present study was supported by the 10.13039/501100004359Swedish Research Council (grant no: 2023-02428), the US Centers for Disease Prevention and Control (grant no: R01TS000324-01-00), and the European Research Council Starting Grant (MegaALS, grant no: 802091).

## Declaration of competing interest

There are no conflict of interest among all authors.

## Data Availability

Data used in the present study are not publicly available due to EU and Swedish regulations. Please contact the corresponding author for more information.
