# Supplementary material for: Immune cells and the trajectories of depression, anxiety, and cognitive function among people with amyotrophic lateral sclerosis
Source: Brain Behav Immun Health. 2024 Nov 22;42:100907. doi: 10.1016/j.bbih.2024.100907 (PMC11625338; doi:10.1016/j.bbih.2024.100907)
Supplement: Multimedia component 1 [file mmc1.docx]

Supplementary Table 1 Correlations between T cell subsets and mental health outcomes using data from first measurements*

|  | Anxiety | | | Depression | | | Cognition | | |
| --- | --- | --- | --- | --- | --- | --- | --- | --- | --- |
| Cell type | estimate | se | *p* | estimate | se | *p* | estimate | se | *p* |
| CD45^+^ | 0.03 | 0.02 | 0.17 | 0.02 | 0.02 | 0.25 | -0.01 | 0.02 | 0.47 |
| CD3^+^ T cells | -0.04 | 0.05 | 0.44 | -0.08 | 0.04 | 0.08 | -0.03 | 0.12 | 0.82 |
| DP | 0.01 | 0.14 | 0.97 | -0.09 | 0.13 | 0.48 | 0.13 | 0.34 | 0.70 |
| DN | 0.41 | 0.39 | 0.30 | 0.09 | 0.35 | 0.79 | -0.04 | 0.04 | 0.35 |
| CD8^+^ T cells | 0.02 | 0.04 | 0.70 | 0.01 | 0.04 | 0.88 | -0.03 | 0.04 | 0.40 |
| Naïve CD8^+^ | 0.03 | 0.03 | 0.33 | 0.02 | 0.03 | 0.64 | 0.00 | 0.03 | 0.92 |
| CD8^+^ T_EMRA_ | -0.02 | 0.02 | 0.37 | -0.03 | 0.02 | 0.21 | 0.00 | 0.02 | 0.84 |
| CD8^+^ T_EM_ | 0.01 | 0.05 | 0.84 | 0.06 | 0.05 | 0.25 | 0.00 | 0.05 | 0.96 |
| CD8^+^ T_CM_ | 0.02 | 0.05 | 0.70 | 0.05 | 0.05 | 0.34 | -0.03 | 0.05 | 0.54 |
| CD4^+^ T cells | -0.02 | 0.04 | 0.65 | 0.00 | 0.03 | 0.98 | 0.03 | 0.03 | 0.42 |
| Naïve CD4^+^ | -0.01 | 0.03 | 0.82 | -0.04 | 0.03 | 0.14 | 0.01 | 0.03 | 0.66 |
| CD4^+^ T_EMRA_ | -0.09 | 0.06 | 0.13 | -0.06 | 0.05 | 0.26 | 0.08 | 0.05 | 0.13 |
| CD4^+^ T_EM_ | 0.03 | 0.05 | 0.53 | 0.04 | 0.05 | 0.42 | -0.06 | 0.04 | 0.19 |
| Th1 _EM_ | 0.01 | 0.03 | 0.86 | 0.00 | 0.03 | 0.86 | -0.02 | 0.02 | 0.43 |
| Th1/Th17 _EM_ | -0.03 | 0.04 | 0.45 | -0.04 | 0.04 | 0.30 | 0.01 | 0.03 | 0.82 |
| Th2 _EM_ | 0.05 | 0.06 | 0.46 | 0.06 | 0.06 | 0.25 | 0.05 | 0.05 | 0.33 |
| Th17 _EM_ | 0.00 | 0.07 | 0.96 | 0.00 | 0.06 | 0.95 | 0.03 | 0.06 | 0.66 |
| CD4^+^ T_CM_ | 0.04 | 0.04 | 0.36 | **0.09** | **0.04** | **0.02** | -0.02 | 0.04 | 0.59 |
| Th1 _CM_ | 0.15 | 0.08 | 0.06 | 0.06 | 0.07 | 0.41 | 0.03 | 0.07 | 0.61 |
| Th1/Th17 _CM_ | 0.04 | 0.07 | 0.57 | -0.02 | 0.06 | 0.75 | -0.04 | 0.06 | 0.50 |
| Th2_CM_ | **-0.15** | **0.07** | **0.05** | -0.02 | 0.07 | 0.81 | 0.03 | 0.06 | 0.68 |
| Th17_CM_ | -0.04 | 0.09 | 0.68 | -0.02 | 0.08 | 0.81 | -0.02 | 0.08 | 0.83 |
| CD4^+^/ CD8^+^ | 0.04 | 0.25 | 0.86 | 0.16 | 0.23 | 0.47 | 0.36 | 0.21 | 0.08 |
| CD4^+^Th1 | 0.08 | 0.06 | 0.20 | 0.08 | 0.05 | 0.13 | -0.07 | 0.05 | 0.15 |
| CD4^+^Th2 | -0.01 | 0.12 | 0.91 | 0.25 | 0.11 | 0.03 | 0.02 | 0.11 | 0.82 |
| CD4^+^Th2 | 0.06 | 0.12 | 0.61 | 0.19 | 0.11 | 0.08 | -0.05 | 0.11 | 0.65 |
| CD4^+^Th1/Th17 | 0.05 | 0.08 | 0.53 | 0.05 | 0.07 | 0.53 | -0.06 | 0.07 | 0.37 |

se: standard error

*Derived from linear regression, adjusted for age at diagnosis, sex, and site of onset.

Supplementary Table 2 Correlations between T cell subsets and mental health outcomes using data from repeated measurements*

|  | Anxiety | | | Depression | | | | Cognition | | | |
| --- | --- | --- | --- | --- | --- | --- | --- | --- | --- | --- | --- |
| Cell type | estimate | se | *p* | estimate | | se | *p* | estimate | se | | *p* |
| CD45^+^ | 0.02 | 0.01 | 0.11 | **0.03** | | **0.01** | **0.04** | -0.02 | 0.01 | | 0.16 |
| CD3^+^ T cells | -0.04 | 0.04 | 0.33 | -0.07 | | 0.04 | 0.07 | -0.02 | 0.03 | | 0.65 |
| DP | -0.03 | 0.13 | 0.85 | -0.09 | | 0.12 | 0.47 | -0.06 | 0.13 | | 0.61 |
| DN | 0.27 | 0.22 | 0.23 | 0.17 | | 0.27 | 0.55 | 0.08 | 0.26 | | 0.77 |
| CD8^+^ T cells | -0.01 | 0.03 | 0.81 | -0.02 | | 0.04 | 0.65 | -0.02 | 0.03 | | 0.51 |
| Naïve CD8^+^ | 0.04 | 0.03 | 0.11 | 0.00 | | 0.03 | 0.92 | 0.01 | 0.03 | | 0.66 |
| CD8^+^ T_EMRA_ | -0.02 | 0.02 | 0.32 | -0.02 | | 0.02 | 0.41 | 0.01 | 0.02 | | 0.66 |
| CD8^+^ T_EM_ | -0.01 | 0.04 | 0.70 | 0.02 | | 0.04 | 0.54 | -0.03 | 0.04 | | 0.43 |
| CD8^+^ T_CM_ | 0.00 | 0.03 | 0.86 | 0.03 | | 0.03 | 0.36 | -0.03 | 0.03 | | 0.31 |
| CD4^+^ T cells | 0.00 | 0.03 | 0.99 | 0.01 | | 0.03 | 0.81 | 0.02 | 0.03 | | 0.54 |
| Naïve CD4^+^ | 0.01 | 0.03 | 0.80 | -0.03 | | 0.03 | 0.28 | 0.02 | 0.02 | | 0.37 |
| CD4^+^ T_EMRA_ | -0.06 | 0.05 | 0.24 | -0.05 | | 0.05 | 0.33 | 0.03 | 0.04 | | 0.45 |
| CD4^+^ T_EM_ | 0.04 | 0.05 | 0.46 | 0.03 | | 0.05 | 0.60 | -0.06 | 0.04 | | 0.14 |
| Th1 _EM_ | 0.02 | 0.02 | 0.22 | 0.03 | | 0.02 | 0.10 | -0.01 | 0.02 | | 0.75 |
| Th1/Th17_EM_ | -0.03 | 0.03 | 0.35 | -0.05 | | 0.03 | 0.16 | 0.01 | 0.03 | | 0.85 |
| Th2_EM_ | -0.01 | 0.03 | 0.83 | -0.04 | | 0.03 | 0.19 | 0.00 | 0.04 | | 0.99 |
| Th17_EM_ | -0.02 | 0.05 | 0.76 | -0.01 | | 0.05 | 0.87 | 0.05 | 0.05 | | 0.35 |
| CD4^+^ T_CM_ | -0.01 | 0.04 | 0.74 | 0.05 | | 0.03 | 0.13 | -0.02 | 0.03 | | 0.44 |
| Th1_CM_ | 0.08 | 0.06 | 0.19 | 0.04 | | 0.06 | 0.52 | 0.06 | 0.06 | | 0.33 |
| Th1/Th17_CM_ | 0.04 | 0.06 | 0.46 | -0.01 | | 0.05 | 0.80 | -0.05 | 0.05 | | 0.28 |
| Th2_CM_ | **-0.15** | **0.07** | **0.02** | -0.01 | | 0.07 | 0.88 | 0.03 | 0.05 | | 0.60 |
| Th17_CM_ | 0.00 | 0.07 | 0.99 | -0.02 | | 0.07 | 0.82 | -0.04 | 0.07 | | 0.51 |
| CD4^+^/ CD8^+^ | 0.16 | 0.24 | 0.51 | 0.17 | 0.20 | | 0.40 | 0.24 | 0.18 | 0.18 | |
| CD4^+^Th1 | 0.05 | 0.05 | 0.25 | 0.09 | 0.05 | | 0.09 | -0.05 | 0.04 | 0.20 | |
| CD4^+^Th2 | -0.13 | 0.09 | 0.17 | 0.02 | 0.10 | | 0.80 | -0.05 | 0.10 | 0.58 | |
| CD4^+^Th2 | -0.02 | 0.10 | 0.81 | 0.10 | 0.10 | | 0.30 | -0.09 | 0.10 | 0.37 | |
| CD4^+^Th1/Th17 | 0.00 | 0.07 | 0.95 | 0.02 | 0.07 | | 0.82 | -0.07 | 0.07 | 0.26 | |

se: standard error

*Derived from linear mixed model with random intercept and random slope, adjusted for age at diagnosis, sex, site of onset, and all parent categories of a specific cell type.

Supplementary Table 3 Association between counts of leukocytes at the time of diagnosis and risk of demonstrating a negative trajectory of mental health outcomes during follow-up – sensitivity analysis after considering CRP levels*

|  | Odds Ratio (95% Confidence Interval) | | |
| --- | --- | --- | --- |
| Cell type | Anxiety | Depression | Cognition |
| Leukocytes | 2.76 (1.14-6.67) | 0.66 (0.33-1.29) | 0.82 (0.37-1.81) |
| Neutrophils | 2.65 (1.04-6.73) | 0.77 (0.39-1.52) | 0.89 (0.41-1.90) |
| Lymphocytes | 1.52 (0.72-3.19) | 0.70 (0.39-1.26) | 0.86 (0.43-1.70) |
| Monocytes | 3.19 (1.05-9.70) | 0.54 (0.23-1.23) | 0.61 (0.23-1.60) |

se: standard error

*Derived from logistic regression, adjusted for age at diagnosis, sex, and site of onset.

Supplementary Table 4 Association between T cell subsets at the time of diagnosis and risk of demonstrating a negative trajectory of mental health outcomes during follow-up*

|  | Odds Ratio (95% Confidence Interval) | | |
| --- | --- | --- | --- |
| Cell type | Anxiety | Depression | Cognition |
| CD45^+^ | 1.10 (0.50-2.43) | 1.21 (0.66-2.20) | 0.82 (0.42-1.60) |
| CD3^+^ T cells | 1.20 (0.44-3.30) | 1.70 (0.72-4.00) | 1.64 (0.74-3.61) |
| DP | 0.91 (0.37-2.28) | 0.98 (0.49-1.95) | 1.12 (0.55-2.28) |
| DN | 0.98 (0.41-2.31) | 1.56 (0.75-3.28) | 1.01 (0.49-2.11) |
| CD8^+^ T cells | 0.69 (0.30-1.56) | 1.22 (0.64-2.33) | 1.47 (0.75-2.89) |
| Naïve CD8^+^ | 1.68 (0.60-4.72) | **0.36 (0.16-0.82)** | 1.02 (0.54-1.93) |
| CD8^+^ T_EMRA_ | 0.47 (0.18-1.21) | 0.59 (0.30-1.18) | 1.67 (0.80-3.46) |
| CD8^+^ T_EM_ | 0.36 (0.11-1.17) | **0.42 (0.20-0.90)** | 1.51 (0.75-3.04) |
| CD8^+^ T_CM_ | 1.04 (0.45-2.37) | **0.45 (0.22-0.95)** | 1.50 (0.76-2.98) |
| CD4^+^ T cells | 1.54 (0.67-3.56) | 0.78 (0.40-1.54) | 0.70 (0.36-1.39) |
| Naïve CD4^+^ | 1.13 (0.48-2.64) | 0.97 (0.50-1.87) | 0.94 (0.50-1.78) |
| CD4^+^ T_EMRA_ | 1.23 (0.53-2.81) | 0.87 (0.44-1.73) | 0.83 (0.40-1.74) |
| CD4^+^ T_EM_ | 0.50 (0.20-1.23) | 0.86 (0.46-1.60) | 1.52 (0.76-3.07) |
| Th1 _EM_ | 1.37 (0.56-3.34) | 1.22 (0.66-2.25) | 1.24 (0.63-2.46) |
| Th1/Th17 _EM_ | 0.84 (0.37-1.88) | 1.05 (0.54-2.07) | 1.28 (0.59-2.77) |
| Th2 _EM_ | 0.45 (0.13-1.61) | 0.58 (0.24-1.39) | 0.97 (0.44-2.15) |
| Th17 _EM_ | 0.95 (0.39-2.36) | 1.01 (0.54-1.90) | 0.51 (0.24-1.07) |
| CD4^+^ T_CM_ | 1.40 (0.55-3.56) | 0.98 (0.48-2.01) | 1.01 (0.49-2.06) |
| Th1_CM_ | 1.04 (0.49-2.19) | 0.74 (0.41-1.32) | 1.53 (0.83-2.82) |
| Th1/Th17_CM_ | 1.05 (0.48-2.30) | 1.15 (0.60-2.22) | 2.29 (0.95-5.54) |
| Th2_CM_ | 0.79 (0.26-2.39) | 1.10 (0.51-2.40) | 0.38 (0.12-1.27) |
| Th17_CM_ | 1.05 (0.46-2.38) | 1.13 (0.65-1.99) | **0.34 (0.13-0.91**) |
| CD4^+^/ CD8^+^ | 1.50 (0.67-3.35) | 0.83 (0.43-1.59) | 0.69 (0.36-1.35) |
| CD4^+^Th1 | 0.84 (0.34-2.11) | 0.92 (0.49-1.76) | 1.53 (0.78-3.02) |
| CD4^+^Th2 | 0.79 (0.28-2.21) | 0.89 (0.44-1.81) | 0.77 (0.32-1.86) |
| CD4^+^Th2 | 1.17 (0.49-2.76) | 1.06 (0.55-2.05) | 0.56 (0.26-1.23) |
| CD4^+^Th1/Th17 | 0.97 (0.45-2.12) | 1.06 (0.55-2.04) | 1.54 (0.76-3.09) |

se: standard error

*Derived from linear mixed model with random intercept and random slope, adjusted for age at diagnosis, sex, and site of onset.
